# What is semantic diversity and why does it facilitate visual word recognition?

**DOI:** 10.3758/s13428-020-01440-1

**Published:** 2020-07-14

**Authors:** Benedetta Cevoli, Chris Watkins, Kathleen Rastle

**Affiliations:** 1 Department of Psychology, Royal Holloway, University of London, Egham, TW20 0EX UK; 2 Computer Science Department, Royal Holloway, University of London, Egham, TW20 0EX UK

**Keywords:** Semantic diversity, Word frequency, Lexical ambiguity, Latent semantic analysis

## Abstract

Previous research has speculated that semantic diversity and lexical ambiguity may be closely related constructs. Our research sought to test this claim in respect of the semantic diversity measure proposed by Hoffman et al. (2013). To this end, we replicated the procedure described by Hoffman et al., Behavior Research Methods, 45(3), 718–730 (2013) for computing multidimensional representations of contextual information using Latent Semantic Analysis, and from these we derived semantic diversity values for 28,555 words. We then replicated the facilitatory effect of semantic diversity on word recognition using existing data resources and observed this effect to be greater for low-frequency words. Yet, we found no relationship between this measure and lexical ambiguity effects in word recognition. Further analysis of the LSA-based contextual representations used to compute Hoffman et al. (2013) measure of semantic diversity revealed that they do not capture the distinct meanings of ambiguous words. Instead, these contextual representations appear to capture general information about the topics and types of written material in which words occur. These analyses suggest that the semantic diversity metric previously proposed by Hoffman et al. (2013) facilitates word recognition because high-diversity words are likely to have been encountered no matter what one has read, whereas many participants may not have encountered lower-diversity words simply because the topics and types of written material in which they occur are more restricted.

**Electronic supplementary material:**

The online version of this article (10.3758/s13428-020-01440-1) contains supplementary material, which is available to authorized users.

Becoming a skilled reader involves the accumulation of experience with individual words. This experience is thought to be encoded in lexical representations and to contribute to word recognition. Most often, we think of lexical experience in terms of word frequency (i.e. the number of times that a word is encountered). It is well known that word frequency is a powerful determinant of word recognition time, with high-frequency words recognised more rapidly than low-frequency words (e.g. Forster & Chambers, 1973; see Brysbaert, Mandera, & Keuleers, 2018; Murray & Forster, 2004 for reviews).

The conceptualisation of lexical experience in terms of word frequency reflects a theoretical commitment about the nature of learning; specifically, that learning is strengthened through repetition. However, recent research has suggested that the accumulation of lexical experience is more nuanced than a simple count of one’s encounters with individual words. Instead, this research suggests that learning may be strengthened by encountering words in a variety of different semantic and syntactic contexts, and hence that some measure of contextual variation may provide a superior conceptualisation of lexical experience (see e.g. Nation, 2017, for discussion).

One means of capturing contextual variation is through a construct known as semantic diversity, described in this journal by Hoffman, Lambon Ralph, and Rogers (2013). The semantic diversity metric proposed by Hoffman et al. (2013) is calculated using latent semantic analysis (LSA; Landauer & Dumais, 1997), and is meant to reflect the average semantic similarity across all of the contexts in which a word occurs. Words high in semantic diversity occur in contexts that have lower similarity to one another than words low in semantic diversity. Previous research shows that this measure of semantic diversity facilitates word recognition in both adults (Hoffman & Woollams, 2015) and children (Hsiao & Nation, 2018; Pagán, Bird, Hsiao, & Nation, 2019) beyond the effect of word frequency.

The original purpose of Hoffman et al.’s (2013) work on the semantic diversity metric was to advance understanding of lexical ambiguity. Most words in English (as for other languages) have multiple interpretations (Rodd, Gaskell, & Marslen-Wilson, 2002). Words that map onto two or more unrelated meanings (e.g. bark) are *homonyms*, while words characterised by multiple related senses (e.g. run) are *polysemes* (Rodd et al., 2002). Research has suggested that polysemous words are recognised faster and more accurately than unambiguous controls, while homonymous words are recognised more slowly and less accurately than unambiguous controls (e.g., Armstrong & Plaut, 2016; Klepousniotou, Titone, & Romero, 2008; Rodd et al., 2002). If variation in contextual usage of a word reflects variation in semantic meaning, then indeed, these constructs might be measuring the same thing.

Hoffman et al. (2013) noted that one problem with the literature on lexical ambiguity is that it conceptualises words as falling into discrete categories (e.g. polysemous, unambiguous) based on the structure of dictionary entries (Klein & Murphy, 2001; Rodd et al., 2002) or subjective ratings (Hino, Lupker, & Pexman, 2002; Pexman, Hino, & Lupker, 2004). They argued that the use of discrete senses or meanings reflects an attempt by lexicographers to segment “*continuous, context-dependent variation*”, and that their semantic diversity metric is preferable because it offers “*an alternative, computationally derived measure of ambiguity based on the assumption that the meanings of words vary continuously as a function of their context*” (pp. 726-727). Subsequent work has continued to postulate a relationship between these constructs; for example, “*the processing advantage for polysemous words in lexical decision might be related to the fact that polysemous words tend to be more semantically diverse*” (Hsiao & Nation, 2018, p. 115).

Despite the appearance of a close relationship between the semantic diversity metric proposed by Hoffman et al. (2013) and lexical ambiguity, we are unaware of any direct evidence for this view. Further, it is important to stress that modelling dynamically changing meaning of words in context is challenging. Hoffman et al.'s (2013) methodology stipulates that the context of a word is the 1000-word section of text in which it occurs, and that the contextual representation of each word is modelled by the entire section of text containing the word. For example, if one section of corpus contains the sentence, “*The elephant played the Moonlight Sonata on the piano*”, then the words *elephant*, *played*, *moonlight*, *sonata*, and *piano* will all have the same contextual vector representation. The semantic content of these words in this specific context is not distinguished by the unique, static, contextual representation provided by this approach. For this reason, we are unsure about whether Hoffman et al.’s (2013) approach can indeed differentiate the nuances of meaning that separate different related usages of polysemous words such as *run*, or different unrelated instances of ambiguous words such as *calf*.

The aim of the present work is to test whether Hoffman et al.’s (2013) conceptualisation of semantic diversity is related to lexical ambiguity as they claimed. To this end, we (a) release materials to compute LSA context vectors and semantic diversity, (b) replicate the previously observed effect of semantic diversity on word recognition in megastudies, and (c) test the relationship between semantic diversity and lexical ambiguity by determining whether semantic diversity accounts for behavioural effects of different types of lexical ambiguity. Following previous work (e.g. Hoffman et al., 2013; Hsiao & Nation, 2018), we derived multidimensional contextual representations of words using LSA, and from these, computed semantic diversity. We then established the semantic diversity advantage on word recognition using data from the English Lexicon Project (ELP; Balota et al., 2007) and British Lexical Project (BLP; Keuleers, Lacey, Rastle, & Brysbaert, 2012) megastudy databases. Finally, we turned to an investigation of whether semantic diversity is able to account for the effects of lexical ambiguity in two high-quality published studies for which materials were available (Armstrong & Plaut, 2016; Rodd et al., 2002). Our analyses suggest that Hoffman et al.’s (2013) measure of semantic diversity could not account for the results of these published studies, and thus we conclude by investigating the nature of information captured through this semantic diversity metric.

## Method

Distributional semantics models propose that a word’s meaning may be derived from the contexts in which it occurs. Words within these models are represented as multi-dimensional vectors, and the distance or angle between vectors provides a measure of their similarity (e.g. Firth, 1957; Landauer & Dumais, 1997; Mikolov, Chen, Corrado, & Dean, 2013). Thus, these models provide a plausible means of characterising the distribution of a word’s meaning in a continuous manner.

**Fig. 1:**
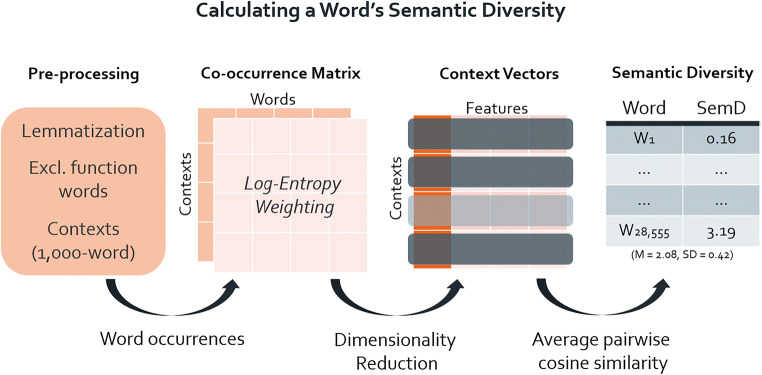
Illustration of semantic diversity procedure

Previous implementations of the semantic diversity metric have used vectors derived from LSA operating on the British National Corpus (Hoffman et al., 2013; Hsiao & Nation, 2018; The British National Corpus, 2007). Although semantic diversity values have been made available previously (Hoffman et al., 2013), we are not aware of any open-access code that would allow psycholinguists to calculate a word’s semantic diversity across different languages and corpora. Thus, a careful replication of the procedure described by Hoffman et al. (2013) for calculating a word’s semantic diversity was conducted (see Fig. 1 for illustration of the procedure). The code implementing these processing steps is available at https://osf.io/xn8u3/.

Our implementation of semantic diversity used the British National Corpus, a collection of 4049 samples of written and spoken British English from a wide range of sources, from newspapers to popular fiction, and comprising 100 million words (British National Corpus Consortium, 2007). Of this, only written documents were selected and then divided into 1000-word contexts. The final chunks from each document were excluded because they may have included less than 1000 words. All non-alphabetic characters (e.g. digits, punctuation) were removed, as well as one-letter words and function words. Finally, any words that appeared fewer than 50 times in the entire corpus and in less than 40 contexts were excluded. These preprocessing steps resulted in 44,477 contexts and 28,555 words, which were used to build a co-occurrence matrix.

The co-occurrence matrix represents contexts in rows and words in columns, and thus reveals the distribution of particular words across different contexts and the clustering of different words in particular contexts. A log entropy weighting was applied to normalise this co-occurrence matrix, and reduced its dimensionality with singular value decomposition (Berry, Dumais, & O’Brien, 1995). This resulted in a set of vectors for each context in the corpus. These vectors, which Hoffman et al. (2013) and subsequent work (e.g. Hsiao & Nation, 2018) referred to as ‘context vectors’, are thought to represent an approximation of the semantic content of each context. Semantic diversity for a particular word is computed by measuring the pairwise cosine similarity between each of the word’s 300-dimensional context vectors, averaging these values, and then applying a log-transform and sign reversal.

Our implementation followed Hsiao and Nation (2018) in using a lemmatised version of the British National Corpus and in excluding function words, while Hoffman et al. (2013) used an inflected version of the same corpus and included function words. Previous literature suggests that these procedural differences should not substantially change the nature of the semantic diversity metric (Hsiao & Nation, 2018) or the performance of semantic vector models (Bullinaria & Levy, 2012). However, in order to make sure that our implementation replicated Hoffman et al. (2013), we also computed semantic diversity using the Hoffman et al. (2013) version of the corpus. The resulting semantic diversity values (using lemmatised and inflected versions of the corpus) correlate highly with each other (*r* = 0.93). Nonetheless, both estimates showed a lower correlation than expected with the semantic diversity measures provided by Hoffman et al. (2013). Specifically, the measures that we computed with the inflected corpus had a correlation of *r* = 0.67 with the ones provided by Hoffman et al. (2013), while the measures computed with the lemmatised corpus had a correlation of *r* = 0.73.

**Fig. 2:**
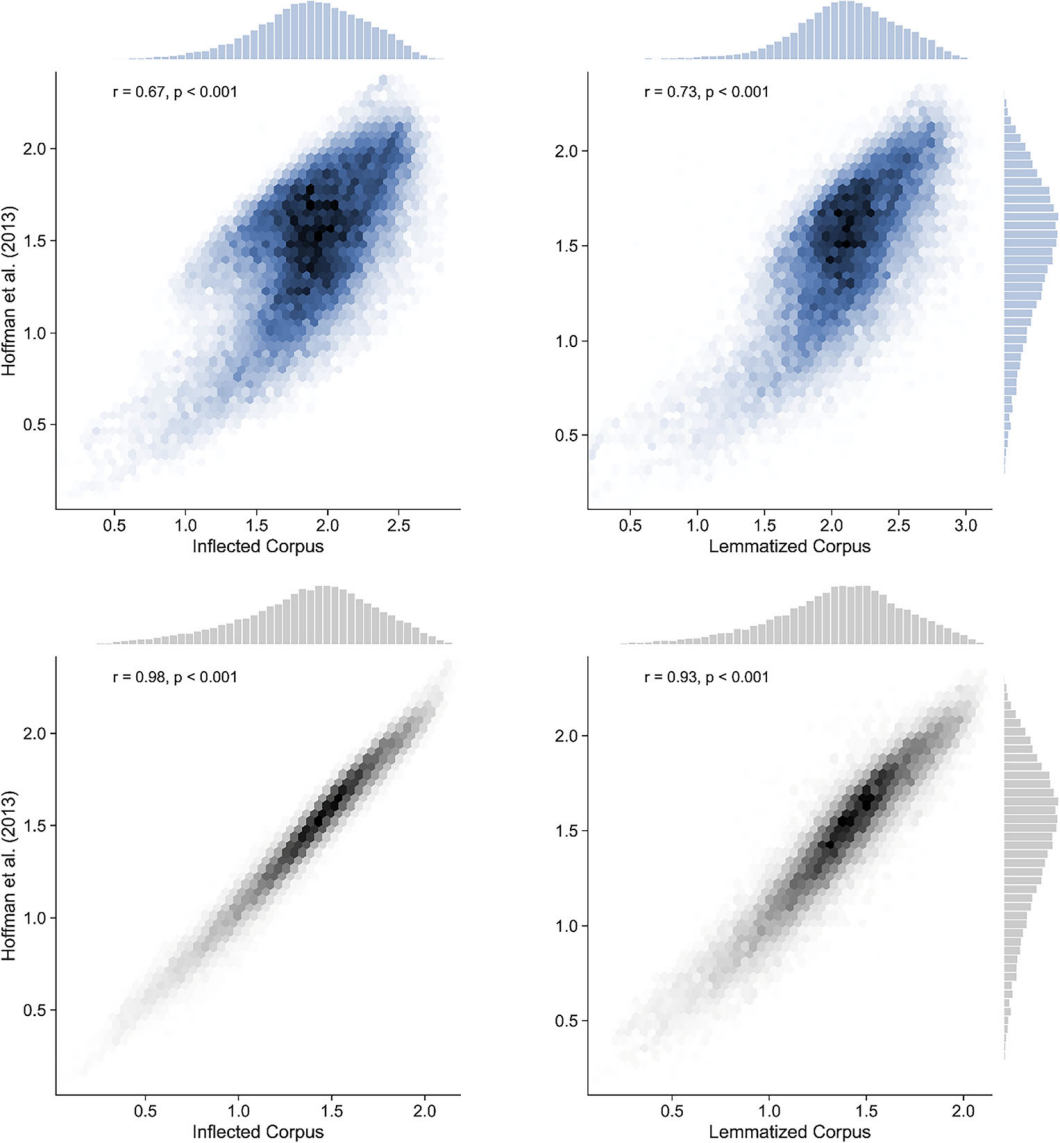
Scatter plots of resulting semantic diversity measures on *x*-axes and the norms reported by Hoffman et al. (2013) and the *y*-axes. On the left, values obtained following the preprocessing procedure described in the methods (lemmatised corpus, exclusion of stop words, etc.), while on the right, values obtained following Hoffman et al. (2013) preprocessing procedure. On the top row (blue) are the measures obtained with the classical output of LSA (weighting by the singular values), while on the bottom row (grey) are the measures obtained without considering the singular values

These correlations are too low given that (a) we replicated the preprocessing procedure described by Hoffman et al. (2013) exactly, and (b) we used the same corpus as Hoffman et al. (2013) for one of the semantic diversity implementations. We cannot provide a definitive explanation for the discrepancy, since the code used by Hoffman et al. (2013) is not available; however, we suspect they did not scale the singular vectors of their co-occurrence matrix by the singular values. Indeed, if we change our implementation to compute semantic space coordinates using unscaled singular vectors, the correlation between our measures and those of Hoffman et al. (2013) increases substantially (*r* = 0.98 for inflected corpus; *r* = 0.93 for lemmatised corpus; see Fig. 2,^1^). Because scaling by the singular values is a key feature of LSA methods, for the remainder of analyses reported in this article, we retained our original semantic diversity measures, computed with the lemmatised corpus and weighted accordingly.

To evaluate the reproducibility of Hoffman et al.’s (2013) procedure for calculating a word’s semantic diversity across different corpora and context lengths, we also computed semantic diversity measures using the English section of the WaCky corpora collection (ukWaC and WaCkypedia corpora, of about 2.8 billion tokens combined; Baroni, Bernardini, Ferraresi, & Zanchetta, 2009) as well as a 100- rather than 1000-word window as context length. We observed strong correlations in semantic diversity values across both context lengths (*r* = .87, *p* < 0.001) and corpora (*r* = .65, *p* < 0.001).

## Results

In order to validate the measures computed following Hoffman et al.’s (2013) procedure, we first replicated previously observed effects of semantic diversity on lexical decision and reading aloud latencies within the English Lexicon Project (ELP; Balota et al., 2007) and the British Lexicon Project (BLP; Keuleers, Lacey, Rastle, & Brysbaert, 2012). The ELP consists of trial-level lexical decision and reading aloud data for 40,481 words collected from 444 participants, while the BLP consists of trial-level lexical decision data for 28,730 words from 78 participants. Semantic diversity measures for 28,555 words were computed following the corpus analysis described in the previous section, while word frequency estimates retrieved were based on the British National Corpus (Van Heuven, Mandera, Keuleers, & Brysbaert, 2014).

We used linear mixed effects models to examine the effect of semantic diversity and its interaction with word frequency on lexical decision and reading aloud data. Analyses were conducted using the lme4 package (Bates, Mächler, Bolker, & Walker, 2015) in R (R Core Team, 2018). Models included semantic diversity, word frequency, and their interaction as fixed effects, while participant and item were included as random intercepts. Trial number was included as a fixed factor. Following Hsiao & Nation (2018), we also controlled for word length and contextual diversity (Adelman, Brown, & Quesada, 2006), as well as age of acquisition (Kuperman, Stadthagen-Gonzalez, & Brysbaert, 2012). We also ran parallel models in which contextual diversity (as indexed by log document count) replaced word frequency (Hsiao & Nation, 2018). To reduce autocorrelation effects from previous trials (Baayen & Milin, 2010), models included fixed effects of previous trial accuracy and latency. Only correct word trials were included in reaction time (RT) analyses, and data points with absolute standardised residuals exceeding 2.5 standard deviations were removed (based on log-transformed RTs; Baayen & Milin, 2010). For visualisation purposes, model estimates were obtained through the package Effects (Fox & Hong, 2009), and transformed RT data were transformed back to raw RTs for ease of interpretation. *P*-values were estimated using the Satterthwaite approximation for degrees of freedom (lmerTest; Kuznetsova, Brockhoff, & Christensen, 2017).

**Fig. 3:**
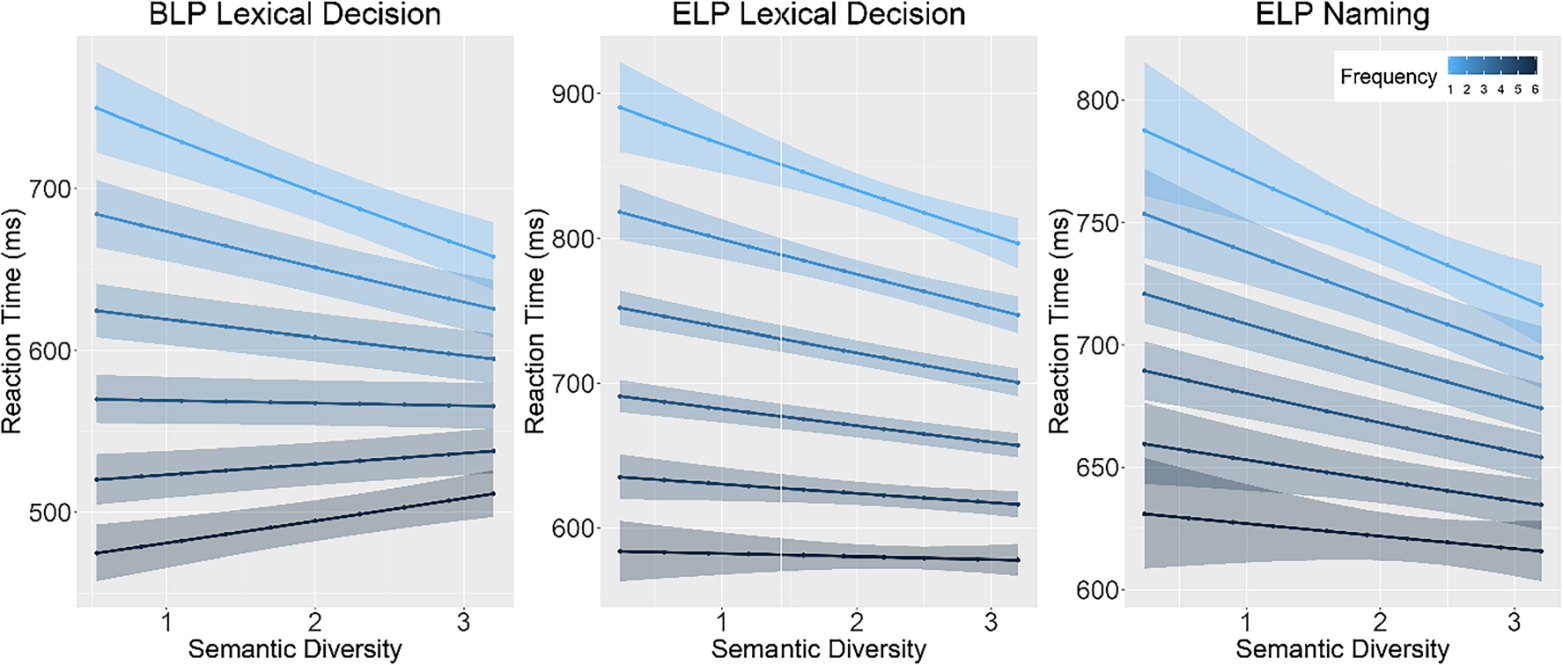
Model estimates of the effect of semantic diversity by frequency on reaction time data as a function of database

Following previous research (Hoffman et al., 2013; Hsiao & Nation, 2018), we observed significant facilitatory effects of both semantic diversity and frequency on reaction time and accuracy across megastudy datasets while controlling for the effects of length and age of acquisition (see Table 1 for summary of results). The significant interaction between frequency and semantic diversity observed on reaction time and accuracy of lexical decision datasets only indicates that the effect of semantic diversity is greater for low-frequency words than for high-frequency words (see Fig. 3). A similar pattern of results was observed when replacing frequency with contextual diversity (indexed by log document count; see supplementary Table 1).

**Table 1:** Summary of results

Dataset	Predictors	Reaction time				Accuracy			
		*b*	*SE*	*t*	*p*	*b*	*SE*	*t*	*p*
BLP Lexical Decision	SemD	< 0.01	< 0.01	−4.03	<0.001	1.11	0.02	5.76	<0.001
	Freq	−0.05	< 0.01	−48.8	<0.001	2.4	0.02	39.87	<0.001
	Length	0.01	< 0.01	8.95	<0.001	1.56	0.02	27.17	<0.001
	AoA	0.04	< 0.01	49.96	<0.001	0.42	0.02	−45.13	<0.001
	SemD*Freq	< 0.01	< 0.01	4.90	<0.001	0.95	0.02	−2.99	0.003
	SemD*Length	< 0.01	< 0.01	−3.54	<0.001	1.07	0.02	3.91	<0.001
	SemD*AoA	< 0.01	< 0.01	2.40	0.016	0.99	0.02	−0.40	0.689
	AoA*Freq	< 0.01	< 0.01	−12.73	<0.001	1.31	0.02	14.69	<0.001
	AoA*Length	< 0.01	< 0.01	1.23	0.219	0.99	0.02	−0.44	0.661
	Length*Freq	< 0.01	< 0.01	1.55	0.121	0.99	0.02	−0.46	0.643
ELP Lexical Decision	SemD	−0.01	< 0.01	−8.32	<0.001	1.15	0.01	11.63	<0.001
	Freq	−0.05	< 0.01	−55.07	<0.001	1.74	0.01	40.56	<0.001
	Length	0.05	< 0.01	69.66	<0.001	1.5	0.01	34.69	<0.001
	AoA	0.06	< 0.01	65.39	<0.001	0.43	0.01	−63.00	<0.001
	SemD*Freq	< 0.01	< 0.01	2.16	0.031	0.96	0.01	−3.64	<0.001
	SemD*Length	< 0.01	< 0.01	3.73	<0.001	0.97	0.01	−2.61	0.009
	SemD*AoA	< 0.01	< 0.01	−2.55	0.011	1.01	0.01	0.45	0.655
	AoA*Freq	−0.01	< 0.01	−11.70	<0.001	1.28	0.01	19.34	<0.001
	AoA*Length	0.01	< 0.01	13.12	<0.001	0.98	0.01	−1.44	0.151
	Length*Freq	< 0.01	< 0.01	−1.63	0.102	0.92	0.01	−6.28	<0.001
ELP Naming	SemD	−0.01	< 0.01	−9.04	<0.001	1.1	0.02	6.43	<0.001
	Freq	−0.02	< 0.01	−27.53	<0.001	1.29	0.02	15.3	<0.001
	Length	0.05	< 0.01	65.19	<0.001	1.08	0.01	5.36	<0.001
	AoA	0.05	< 0.01	61.8	<0.001	0.44	0.02	−50.41	<0.001
	SemD*Freq	< 0.01	< 0.01	1.54	0.125	1.00	0.01	−0.24	0.81
	SemD*Length	< 0.01	< 0.01	−1.34	0.180	0.99	0.01	−1.05	0.295
	SemD*AoA	< 0.01	< 0.01	1.58	0.114	0.99	0.02	−0.85	0.397
	AoA*Freq	−0.01	< 0.01	−12.94	<0.001	1.26	0.02	15.07	<0.001
	AoA*Length	0.01	< 0.01	17.10	<0.001	0.87	0.01	−9.12	<0.001
	Length*Freq	< 0.01	< 0.01	−2.36	0.018	0.96	0.02	−2.20	0.028

We next turned to investigate the relationship between lexical ambiguity and semantic diversity. We selected two prominent studies reporting differences in processing polysemous and homonymous word compared to unambiguous controls in visual lexical decision (Armstrong & Plaut, 2016; Rodd et al., 2002), and sought to replicate these using response time and accuracy measures from the BLP and ELP lexical decision data, and then using our newly computed semantic diversity measures.

### Simulation 1 – Rodd et al. (2002)

Stimuli were selected from two experiments of Rodd et al. (2002), one of the first visual lexical decision studies reporting contrasting effects of polysemy and homonymy on lexical decision performance. Based on the structure of dictionary entries, Rodd et al. (2002) used the number of a word’s meanings and senses as proxies of homonymy and polysemy, respectively.

**Fig. 4:**
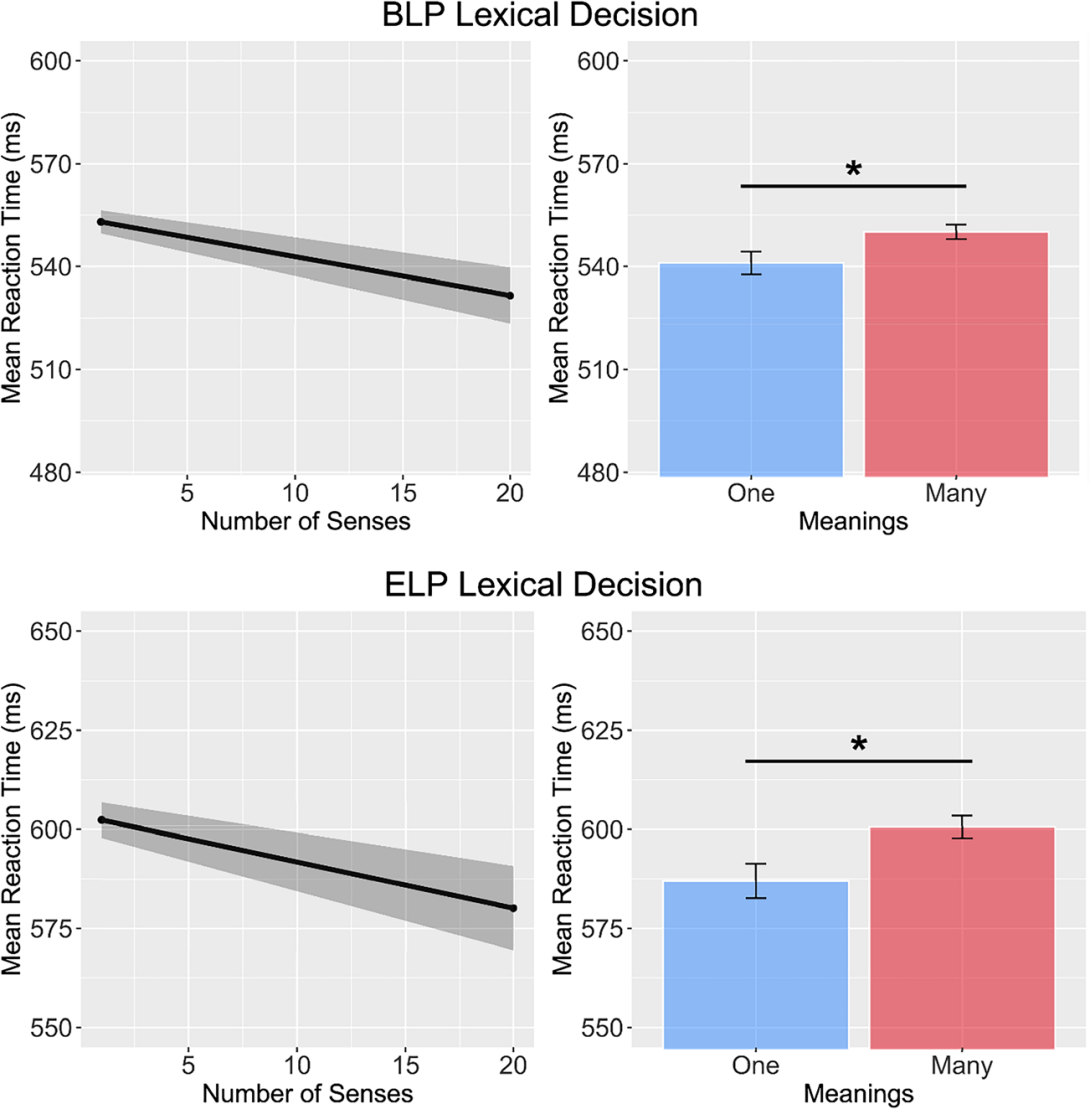
Results of the simulation analysis of Experiment 1 of Rodd et al. (2002) on reaction time data of BLP and ELP. Both datasets show that increasing number of senses speeds performance, while increasing number of meanings slows performance

**Fig. 5:**
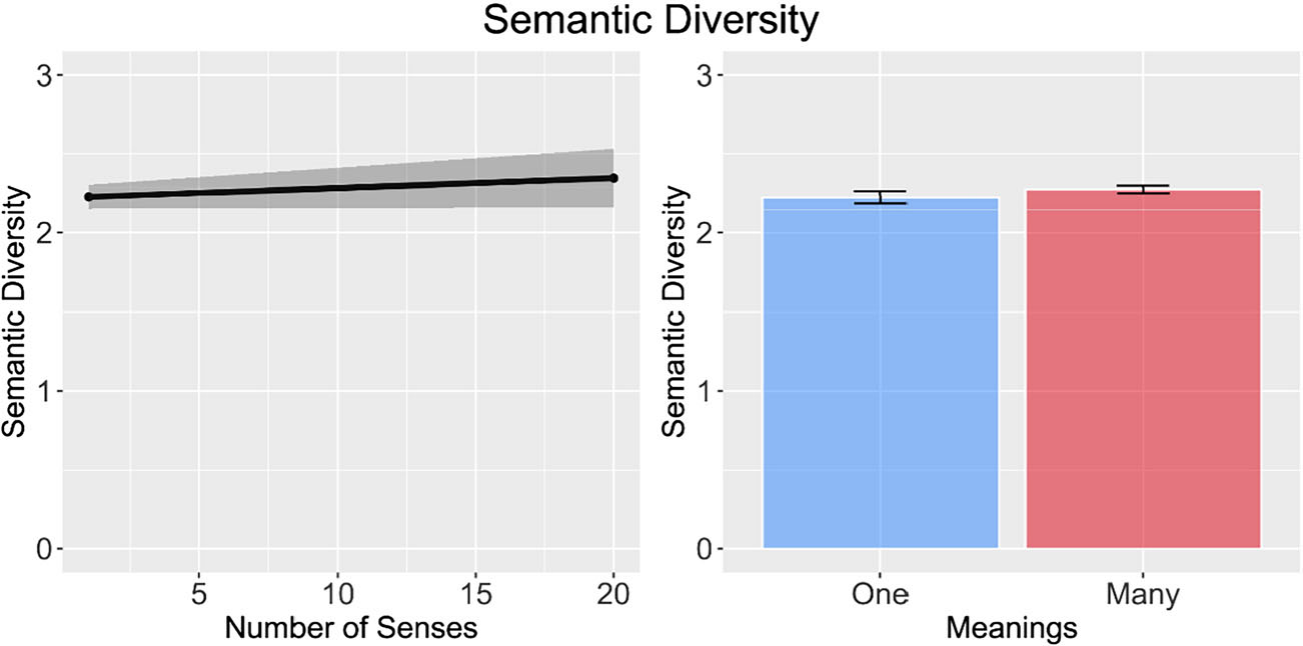
Results of the simulation analysis of Experiment 1 of Rodd et al. (2002) showing no difference in semantic diversity for words with many or few senses and meanings

In their first experiment, Rodd et al. (2002) used a regression design to investigate the impact of multiple meanings and multiple senses on word recognition. They observed that word recognition was slowed when words were characterised by multiple meanings but speeded when words were characterised by multiple senses. This combination of results is also observed in the BLP (number of meanings, *β* = 0.02, *SE* = 0.01, *t* = 1.97, *p* < 0.05; number of senses, *β* =  − 0.01, *SE* < 0.01, *t* =  − 2.23, *p* < 0.05) and in the ELP (number of meanings, *β* = 0.03, *SE* = 0.01, *t* = 2.5, *p* < 0.05; number of senses, *β* =  − 0.01, *SE* < 0.01, *t* =  − 1.96, *p* < 0.05; see Fig. 4). However, our analyses revealed that these effects could not be ascribed to semantic diversity. Semantic diversity values did not differ for these items on number of meanings (*β* = 0.05, *SE* = 0.04, *t* = 1.06, *p* = 0.29) or number of senses (*β* = 0.02, *SE* = 0.02, *t* = 0.95, *p* = 0.34; see Fig. 5).

**Fig. 6:**
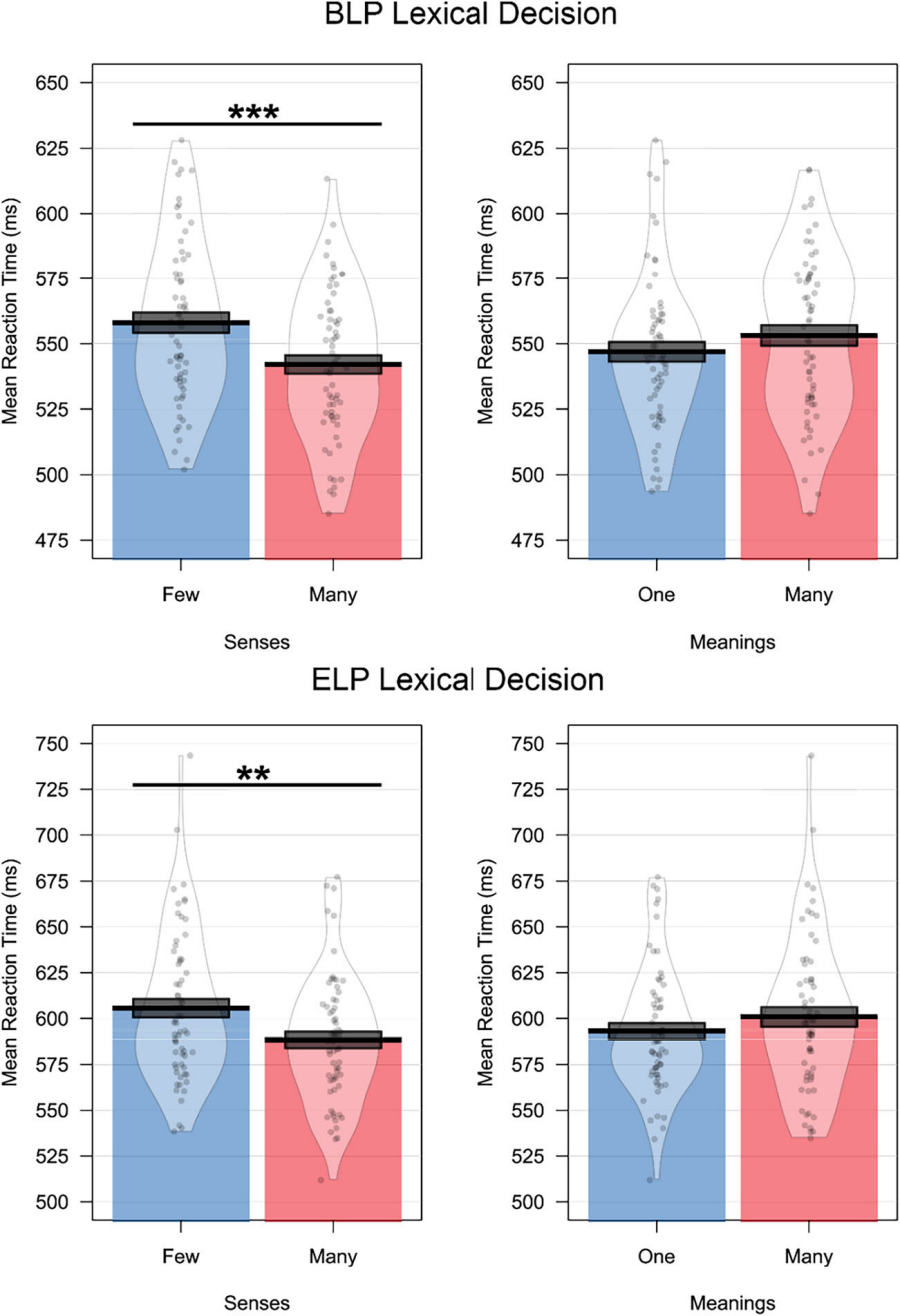
Results of the simulation analysis of Experiment 2 of Rodd et al. (2002) on response time data from the BLP and ELP. Data show that an increased number of senses speeds lexical decision latency, but that there is no effect of the number of meanings

**Fig. 7:**
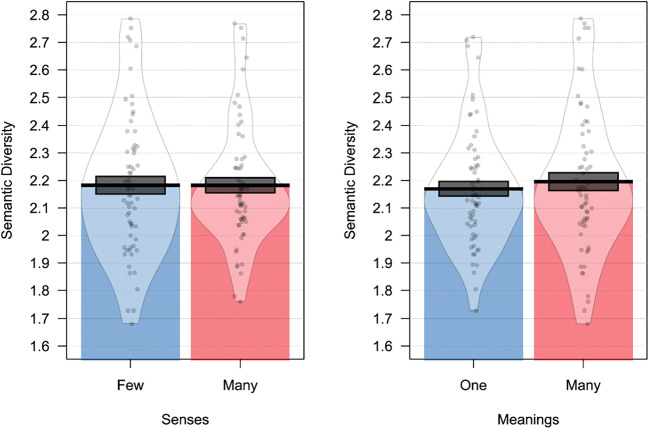
Results of the simulation analysis of Experiment 2 of Rodd et al. (2002) showing no difference in semantic diversity values for words with many or few senses or meanings

In a second experiment, Rodd et al. (2002) used a factorial design manipulating number of senses and number of meanings, and reported a significant effect only of the former. Following the statistical analysis pipeline reported in Rodd et al. (2002), lexical decision data from the BLP and ELP revealed a significant main effect of the number of senses on response time (BLP:*F*_1_(1, 4735) = 18.86, *p* < 0.001; *F*_2_(1,121) = 12.27, *p* < 0.001; *∆RT* = 15 *ms*; ELP: *F*_1_(1, 3763) = 7.23, *p* < 0.01; *F*_2_(1,121) = 6.33, *p* < 0.05; *∆RT* = 16 *ms*; see Fig. 6). There was no effect of number of meanings in the BLP (*F*_1_(1, 4735) = 3.5, *p* = 0.06; *F*_2_(1,121) = 3.2, *p* = 0.07; *∆RT* = 8 *ms*) or the ELP (*F*_1_(1, 3763) = 2.99, *p* = 0.08; *F*_2_(1,121) = 2.22, *p* = 0.13; *∆RT* = 8 *ms*). Once again, while Rodd et al.’s (2002) data were perfectly captured in the BLP and ELP, the pattern reported could not be ascribed to semantic diversity. Semantic diversity values did not differ for number of senses (*F*(1,121) < 0.001, *p* = 0.99) or number of meanings (*F*(1,121) = 0.38, *p* = 0.53) for these items (see Fig. 7).

### Simulation 2 – Armstrong and Plaut (2016)

Armstrong and Plaut (2016) investigated whether the polysemy advantage and homonymy disadvantage found in visual lexical decision are modulated by task difficulty and stimulus contrast. Task difficulty was manipulated by varying the wordlikeness of nonwords in the lexical decision task. Armstrong and Plaut (2016) observed that the polysemy advantage reduced while the homonymy disadvantage increased as task difficulty increased. The authors argued that these findings may help us to understand why in standard lexical decision tasks (which usually correspond to the lower task difficulty condition), the homonymy disadvantage is weak or completely absent, while the polysemy advantage is consistently reported (see Armstrong & Plaut, 2016 for discussion and Eddington & Tokowicz, 2015 for a review of ambiguity literature). Thus, a similar pattern of results is expected to be found in lexical decision data of the BLP and ELP.

**Fig. 8:**
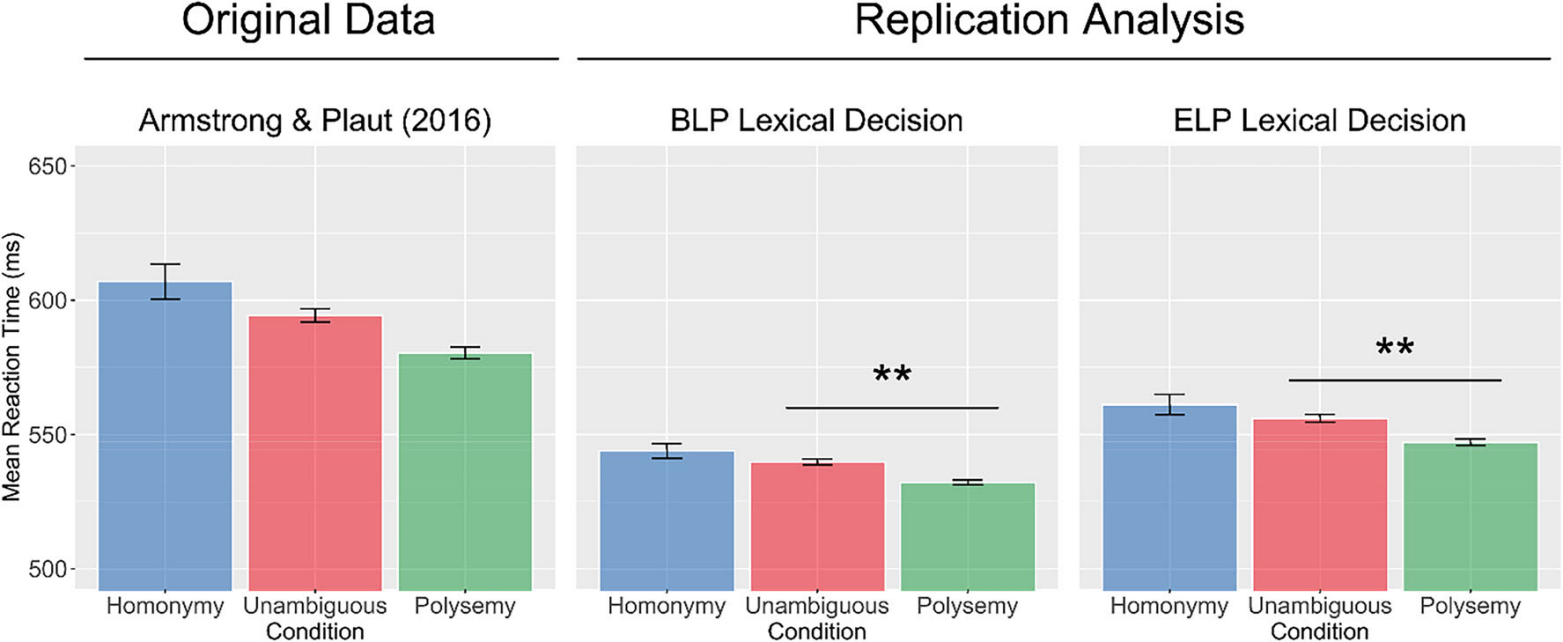
Descriptive bar plots of response time data (left) by type of ambiguity (pooled between all experimental conditions) as reported by Armstrong & Plaut (2016) and bar plots of replication analysis of BLP and ELP (middle and right, respectively) showing a polysemy advantage but no homonymy disadvantage

**Fig. 9:**
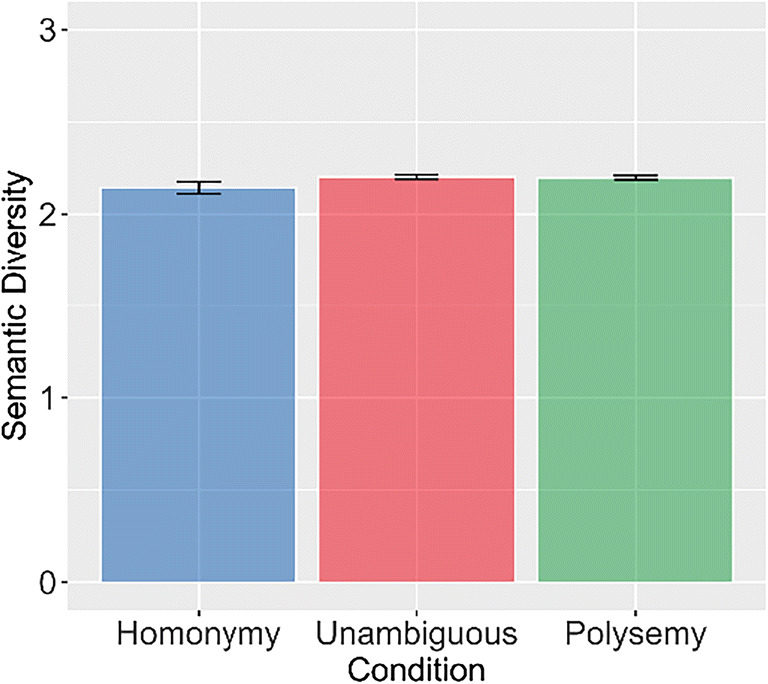
Results of the simulation analysis of Armstrong & Plaut (2016) on semantic diversity measures showing no difference across ambiguity type

Results from our analysis of the BLP and ELP also showed faster recognition of polysemous words relative to unambiguous controls (BLP: *b* = 0.02, *SE* < 0.01, *t* =  −2.66, *p* < 0.01; ELP: *b* =  − 0.02, *SE* = 0.01, *t* =  − 2.70, *p* < 0.01). No significant difference was observed between homonymous and unambiguous words (BLP: *β* < 0.01, *SE* < 0.01, *t* = 1.33, *p* = 0.18; ELP: *b* <0.01, *SE* < 0.01, *t* =   1.41, *p* = 0.16; see Fig. 8). However, again none of these effects are observed in the semantic diversity measures. There was no significant difference in semantic diversity between unambiguous and polysemous words (*β* < 0.01, *SE* = 0.01, *t* =  − 0.37, *p* = 0.7), and while there was a near-significant difference in semantic diversity between unambiguous and homonymous words (*β* =  − 0.02, *SE* < 0.01, *t* =  − 1.93, *p* = 0.05), it was in the opposite of the predicted direction (see Fig. 9).

To evaluate the reproducibility of these results across different corpora and context lengths, we simulated these studies using semantic diversity measures calculated using the ukWaC and WaCkypedia corpora (Baroni et al., 2009), and for 100- rather than 1000-word contexts. These results revealed the same pattern of results as described above for the 100-word contexts, and for the ukWaC corpus. However, semantic diversity measures calculated with the WaCkypeda corpus did show a significant effect of polysemy, with polysemous words showing greater semantic diversity than unambiguous words in both datasets (see Supplementary Table 2 and Supplementary Figure 1). We reserve interpretation of this surprising pattern of results for the Discussion.

In summary, though the effects of polysemy and homonymy reported by Rodd et al. (2002) and Armstrong and Plaut (2016) were also observed in the BLP and ELP using Hoffman et al.’s (2013) model parameters, there was no evidence that these effects could be ascribed to semantic diversity. This result is inconsistent with the claim that semantic diversity provides a continuous measure of the multiple senses and meanings with which words are used in different contexts (Hsiao & Nation, 2018; Hoffman et al., 2013). In the discussion, we consider more fully what semantic diversity is and why it facilitates visual word recognition.

## Discussion

Previous research has proposed that semantic diversity and lexical ambiguity are closely related (Hoffman et al., 2013). However, our analyses suggest that the LSA-based measure of semantic diversity developed by Hoffman et al. (2013) does not capture differences between homonymous, polysemous and unambiguous words. These results may suggest that these different forms of words are not characterised by differences in contextual variation, although this seems unlikely (e.g. that *bank* would not be characterised by greater contextual variation than *perjury*). The other possibility is that the LSA-based measure of semantic diversity described by Hoffman et al. (2013) does not capture this contextual variation. Yet, if this is the case, then it is unclear what their measure of semantic diversity is capturing or why it facilitates word recognition.

One potential explanation is that, as a measure of central tendency, Hoffman et al.’s (2013) conceptualisation of semantic diversity does not reflect the *distribution* of a word’s contexts, and consequently is unable to differentiate between ambiguous and unambiguous words. That is, it may be that the context vectors of ambiguous words such as *bank* show greater variation than those of unambiguous words, but that the averaging process in the calculation of semantic diversity masks this variation.

However, it is also possible that the context vectors themselves are insensitive to the contextual meanings of words. LSA has been used extensively as a topic model for organising and summarising large collections of written text by automatically identifying abstract topics (text classification purposes and recommender systems; Evangelopoulos, Zhang, & Prybutok, 2012; Landauer et al., 2007). However, much less is known about the extent to which LSA captures the contextual nature of semantic content of individual words within the context. Therefore, the nature of information represented within these context vectors requires exploration.

To investigate these possibilities, we selected three examples of highly ambiguous words from Rodd et al. (2002), *calf*, *mole*, and *pupil*, and manually labelled a random 50% of the contexts in which each word occurred within the corpus used to derive our context vectors. For the word *calf*, for example, we decided whether each occurrence related to an animal, a body part, or some other meaning. We then visualised the labelled context vectors using the t-Distributed Stochastic Neighbour Embedding (t-SNE) technique for dimensionality reduction (Van Der Maaten & Hinton, 2008). By visualising the contexts in this manner, we sought to determine whether (a) the context vectors do indeed capture contextual variation but the averaging within the semantic diversity metric fails to reflect this, or (b) the context vectors are insensitive to this semantic variation.

**Fig. 10:**
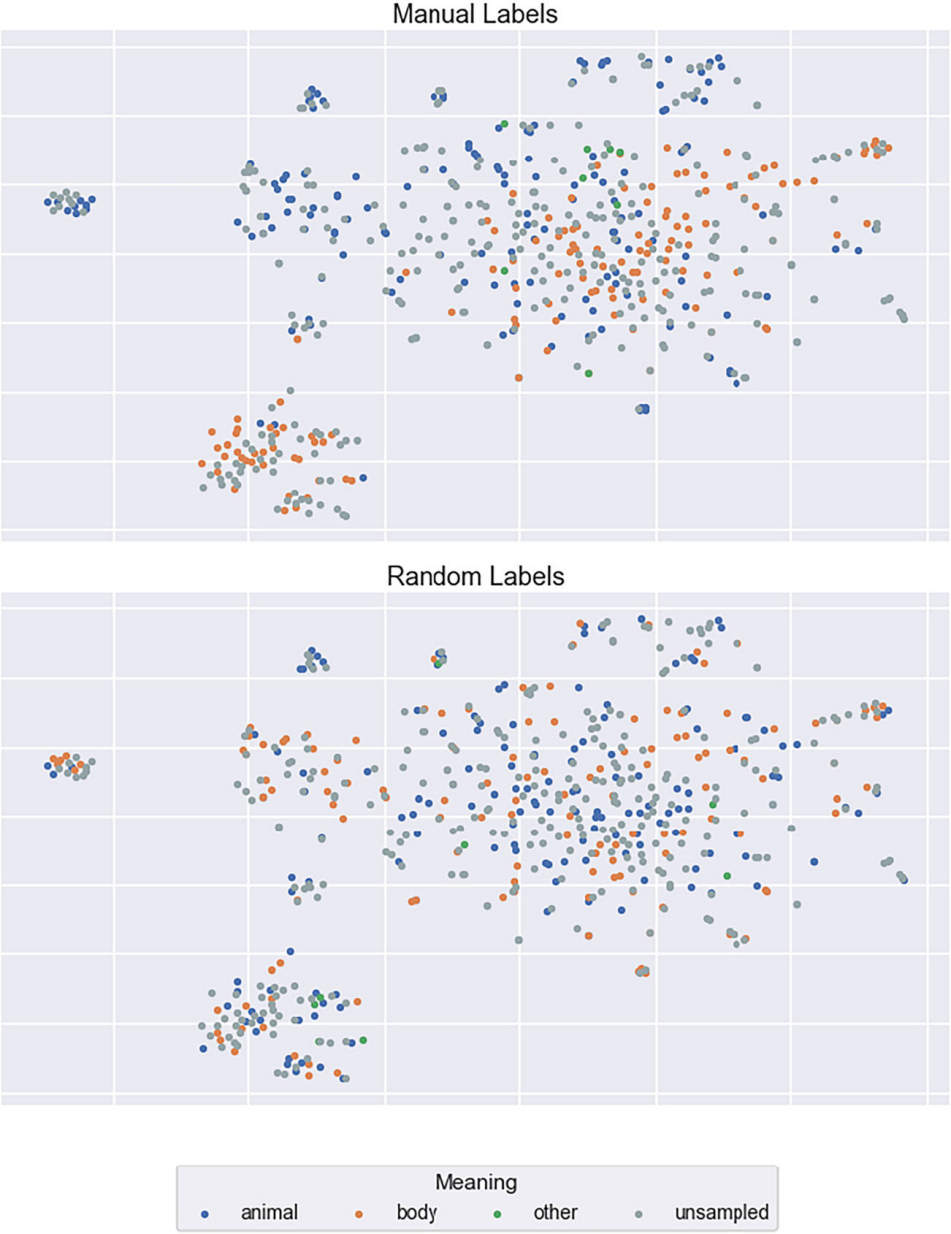
t-SNE plots of the context vectors in which the word *calf* occurs

**Fig. 11:**
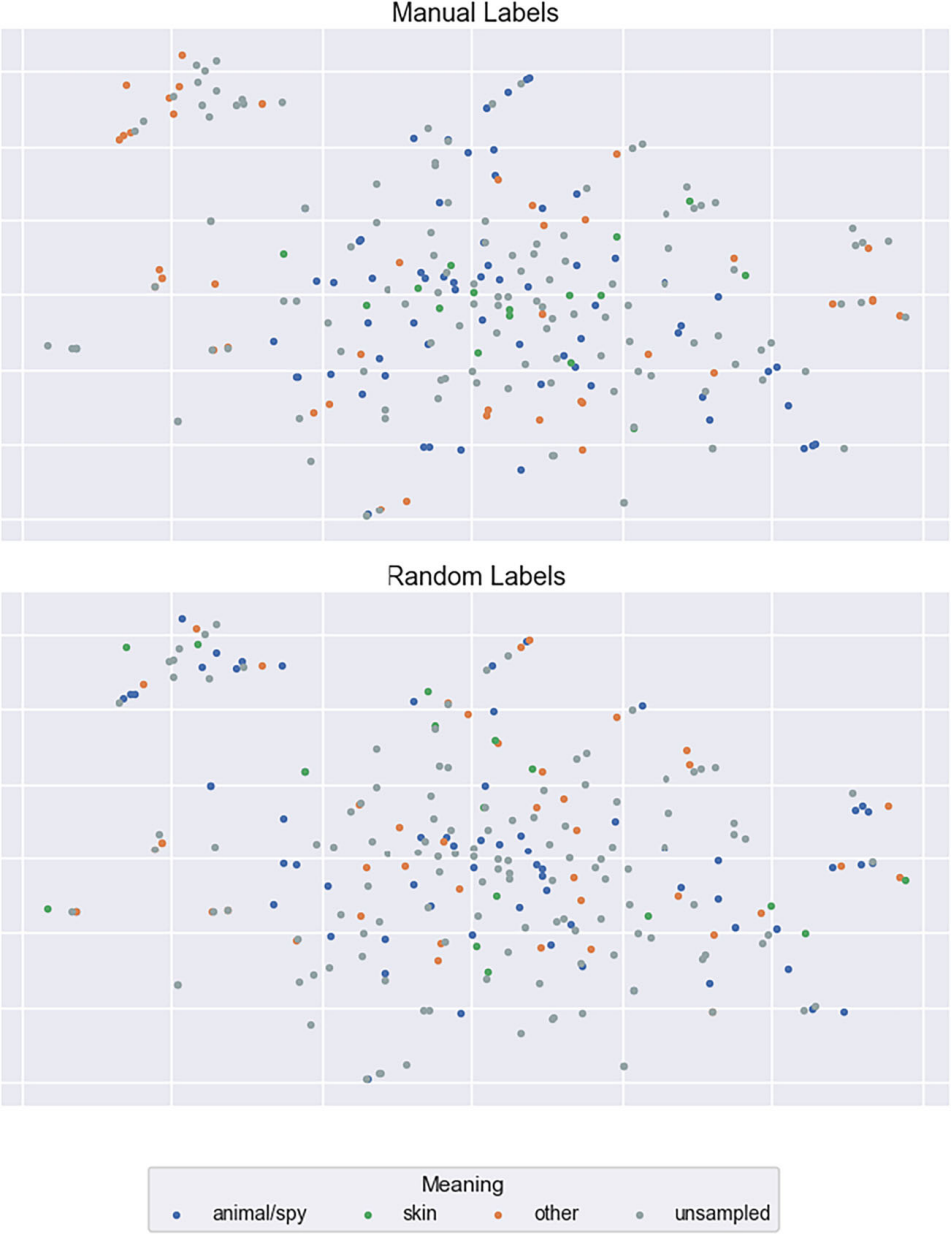
t-SNE plots of the context vectors in which the word *mole* occurs

**Fig. 12:**
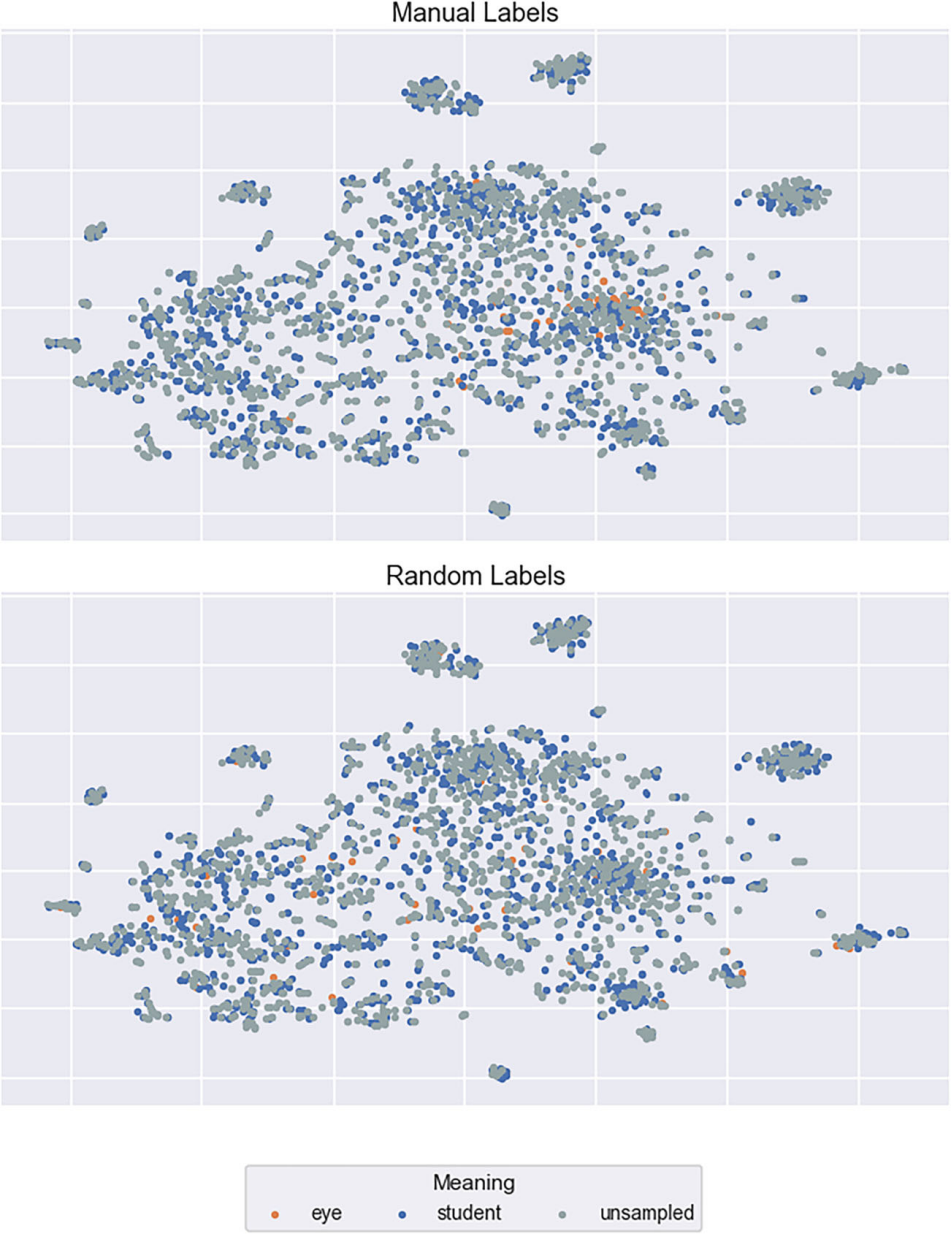
t-SNE plots of the context vectors in which the word *pupil* occurs

It is immediately apparent from Fig. 10 that the LSA context vectors of the word *calf* are not represented in distinct clusters (as would be expected due to its unrelated meanings), but are instead spread widely across the semantic space. The same pattern holds for the distinct meanings of *mole* and *pupil* (Figs. 11 and 12). To assess quantitatively whether there is evidence that the context vectors are representing distinct semantic clusters in the multidimensional LSA space, we computed a Calinski-Harabasz score for each sample word. This score reflects variance between and within clusters; higher scores indicate superior goodness of fit with defined clusters (Caliñski & Harabasz, 1974). Relatively low scores were found for all three examples (3.28, 2.08, and 4.43 for *calf*, *mole*, and *pupil*, respectively). These are similar to the scores derived when the same 50% of contexts were assigned the three possible labels randomly (0.92, 1.13, and 0.97, respectively). These data suggest that LSA-based context vectors are not sensitive to the contextual meanings of ambiguous words, and thus the failure to capture lexical ambiguity effects with the semantic diversity metric lies in the modelling approach itself. This conclusion is consistent with other work showing that LSA fails to identify ambiguity effects compared to other models (Beekhuizen, Milic, Armstrong, & Stevenson, 2018) as well as to understand the contextual meaning of semantically ambiguous words in context (Jamieson, Avery, Johns, & Jones, 2018). Indeed, this outcome raises the important question of what information is captured by LSA context vectors, and why this metric appears to facilitate word recognition.

**Fig. 13:**
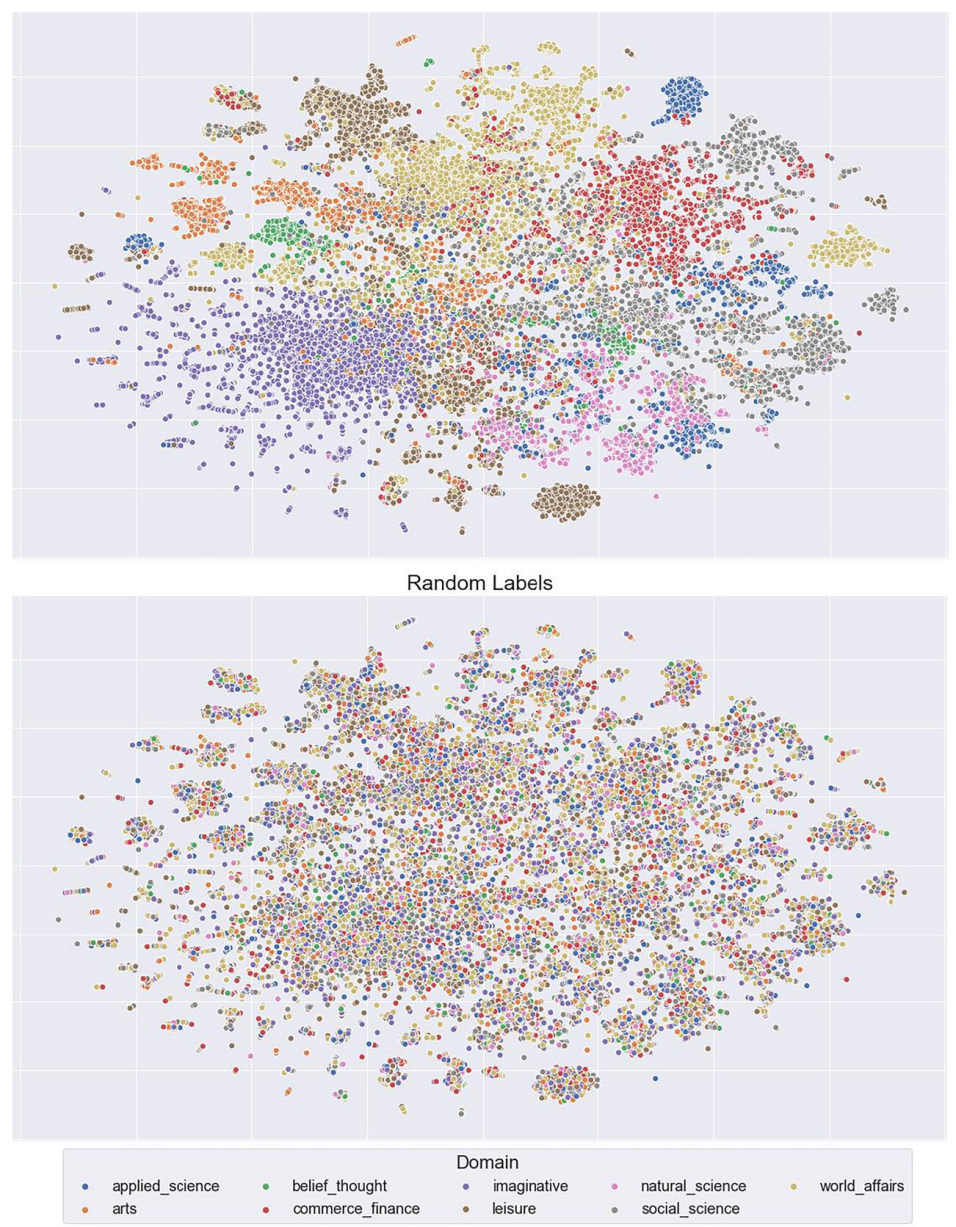
t-SNE plots of the whole corpus labelled by domain on the top (variance ratio: 320.74), while on the bottom are the same labels randomly assigned for comparison (M = 1.00, SD = 0.04 for 1000 iterations)

**Fig. 14:**
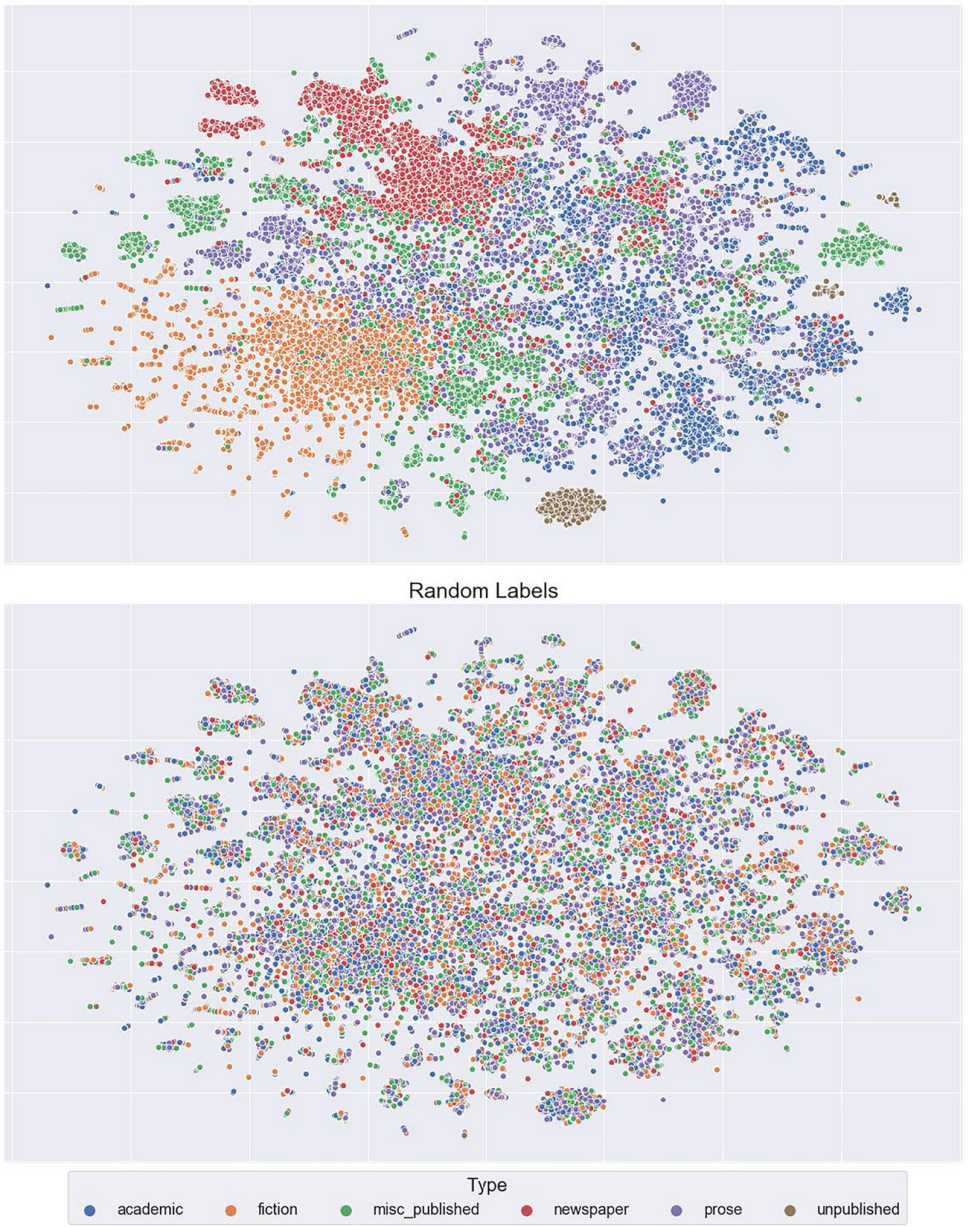
t-SNE plots of the whole corpus labelled by type of written material on the top (variance ratio: 301.12), while on the bottom are the same labels randomly assigned for comparison (M = 1.00, SD = 0.05 for 1000 iterations)

To understand more fully what LSA context vectors represent, we labelled every context within our corpus using metadata pertaining to the general domain of the contexts (e.g. natural science, world affairs) and the type of written material in which the contexts appear (e.g. fiction, newspaper). These data are visualised in Figs. 13 and 14. It is apparent that contexts cluster well along these dimensions. This is confirmed by the Calinski-Harabasz scores comparing clustering based on general domain (320.74) relative to random allocation (*M* = 1.00, *SD* = 0.04 for 1000 iterations), and clustering based on type of written material (301.12) relative to random allocation (*M* = 1.00, *SD* = 0.05 for 1000 iterations).^2^ These data suggest that the LSA context vectors are capturing general properties about how words occur in a corpus, but not capturing information about the nature of word meaning.

It is worthwhile now to consider why simulation analyses using the WaCkypedia corpus revealed that polysemous words had higher semantic diversity than unambiguous words (see Supplementary Table 2 and Supplementary Figure 1). The WaCkypedia corpus is highly constrained compared to the British National Corpus used by Hoffman et al. (2013). It contains text from Wikipedia articles only; that is, topic-constrained texts typical of encyclopaedias with no variation in style or genre. The way that words occur in this corpus may also differ from less constrained formats; for example, the word *film* arises 51 times in the Wikipedia entry for *photography*. To investigate why this corpus performed differently from the British National Corpus, we computed LSA context vectors for the word *flash*, as this was the polysemous word that increased most in semantic diversity when using the WaCkypedia corpus. We wanted to know whether the LSA context vectors would now show clustering based on the different senses of the word *flash*. However, analyses revealed that this was not the case. Instead, the clustering of LSA context vectors was based on *topics* within the WaCkypedia corpus: for example, types of guns, type of ships, movies, rock bands, video games, names of tropical storms, and brands of cameras. This preliminary analysis leads us to believe that the polysemous words used in Rodd et al. (2002) and in Armstrong and Plaut (2016) may occur in more Wikipedia topics than unambiguous words. However, our general conclusions that LSA context vectors capture how words occur in a corpus rather than variations in the nature of word meaning still stand.

Overall, our analyses lead us to suggest that the metric defined by Hoffman et al. (2013) is a measure of a word’s spread across topics and types of contexts, rather than a measure of the diversity of a word’s contextual meaning. This metric is insensitive to the diversity of a word’s meanings; instead, it captures general information about the range of reading situations in which a word might be encountered. Words that are high in Hoffman et al.’s (2013) semantic diversity metric are well-distributed across topics and types of contexts, while words that are low in this semantic diversity metric are specific to particular contexts. Thus, we propose that this metric should instead be referred to as *textual diversity*.

The term textual diversity is intended to provide a more accurate description of the metric proposed by Hoffman et al. (2013), while clearly differentiating it from related constructs. We would argue that the term semantic diversity is inappropriate for Hoffman et al.’s (2013) metric because this metric does not reflect semantic variation of words in context. Instead, we believe that the term textual diversity captures the very nature of Hoffman et al.’s (2013) metric: variation across types of written texts. Nevertheless, it is also important to distinguish textual diversity from contextual diversity (Adelman et al., 2006). The term contextual diversity (as described by Adelman et al., 2006) refers to the count of unique documents in which a word occurs, while textual diversity considers the similarity of their content. If a word occurs in a large number of documents yet covering very similar topics, it will have a high contextual diversity but a low textual diversity.

This proposal may have theoretical implications for understanding the beneficial effect of Hoffman et al.’s (2013) notion of semantic diversity on word recognition. We suggest that textual diversity is related to the probability that a reader will have encountered a word at all. It is for this reason that it facilitates word recognition particularly for low-frequency words. Words with high textual diversity (e.g. ‘diverge’) are spread across topics and types of material and will therefore be encountered irrespective of what is read; in contrast, words with low textual diversity (e.g. ‘crampon’) arise only in specific topics or types of material, and therefore some readers may almost never encounter them if they do not read about these specialised topics. Each low textual diversity word may appear very rare to some fraction of readers who have not read material where the word occurs, and some readers may have never encountered the word at all. In word-recognition paradigms, therefore, performance on such a word will suffer when averaged across readers simply because some readers have rarely encountered it. The impact of textual diversity may be less relevant for high-frequency words, since these are likely to be encountered by nearly all readers, even if they only occur in specific types of texts.

In accordance with modern theoretical accounts of language processing and learning, this proposal identifies distributional properties of words as playing a central role in reading. For example, the expected probability of encountering a word in a given context determines priors that influence word recognition according to the Bayesian reader theory (Norris, 2006, 2009). From a different perspective, the association strength between a word and its contextual usages is pivotal in the naïve discriminative learning theory (Baayen, 2011; Baayen, Chuang, Shafaei-Bajestan, & Blevins, 2019; Hollis, 2019). Moreover, recent evidence suggests that as people accumulate experiences of the world, their vocabulary grows into a richer and more specialised lexicon that usually comprises more uncommon words (Ramscar, Hendrix, Love, & Baayen, 2014; Ramscar, Hendrix, Shaoul, Milin, & Baayen, 2013; Ramscar, Sun, Hendrix, & Baayen, 2017). An interesting prediction that follows from this work is that the influence of textual diversity may change over individuals' lifetime as well reflecting shifts in the nature of their reading experience.

It is also important to note that the findings observed in this paper are circumscribed to the LSA-based approach for calculating a word’s semantic diversity proposed by Hoffman et al. (2013), and thus they may not necessarily apply to other approaches for modelling contextual variation of lexical items (see Jones, Johns, & Recchia, 2012; McDonald & Shillcock, 2001). For example, though both Jones et al. (2012) and Hoffman et al. (2013) conceptualised their work under the term ‘semantic diversity’, there are many methodological differences between these approaches. Whereas Hoffman et al.’s (2013) implementation defines semantic diversity as a context-to-context calculation by comparing contextual representations with each other, Jones et al. (2012) use a word-to-context calculation where words and contexts representations are compared (Johns, Dye, & Jones, 2016a; Johns, Gruenenfelder, Pisoni, & Jones, 2012; Jones, Dye, & Johns, 2017; Jones et al., 2012). Moreover, Jones et al. (2012) sought to produce a measure that replaces word frequency, while Hoffman et al.’s (2013) metric has been shown to contribute lexical processing beyond the effect of word frequency (Hsiao & Nation, 2018; Pagán et al., 2019).

The growing body of literature that investigates the influence of experience in lexical processing using corpus-based models is clearly moving toward a graded conceptualisation of the contextual meanings of words (Rodd, 2020). Ultimately, though we have shown the original work of Hoffman et al. (2013) to be flawed, we agree with their initial proposition that the characterisation of lexical ambiguity based on discrete numbers of dictionary definitions presents severe limitations. Future work should move away from this strict definition of ambiguity in favour of a data-driven approach where it is possible to consider graded overlap between word meaning representations, as well as to measure the dispersion of these representations (Beekhuizen et al., 2018). However, this work must be accompanied by deep analysis of the nature of information being captured through these corpus-based approaches. Our work demonstrates that the LSA-based approach as proposed by Hoffman et al. (2013) is not the appropriate tool for this task. The field of natural language processing has seen exceptionally rapid development in the past twenty years, providing a variety of state-of-art techniques that might be more suitable for modelling the distribution of the semantic contents of individual words (Young, Hazarika, Poria, & Cambria, 2017). Future work using more up-to-date models has the potential to capture contextual variation across different words, and ultimately to help us to understand more deeply the nature of lexical experience.

To summarise, we sought to investigate the relationship between the semantic diversity measure described by Hoffman et al. (2013) and lexical ambiguity. We implemented LSA-based context vectors from which we derived their semantic diversity metric, and we demonstrated that this metric is associated with the speed of word recognition and reading aloud as previously observed in the literature. Despite Hoffman et al.’s (2013) original claims that their measure of semantic diversity and lexical ambiguity are closely related, we found no evidence that effects of lexical ambiguity on word recognition could be ascribed to semantic diversity. Further analysis of the LSA-based context vectors used to derive their semantic diversity metric revealed that they do not sensitively capture information about the different contextual meanings of individual words, and the measure appears instead to encode more general information about the manner in which words occur within a corpus. Thus, we proposed the term *textual diversity* as a better fit for describing the semantic diversity metric defined by Hoffman et al. (2013). These findings have important theoretical implications for understanding why this metric facilitates word recognition.

## Electronic supplementary material

ESM 1 (DOCX 3581 kb)

## Footnotes

1 If our speculation is accurate, then this is indeed an unfortunate error in the original report of Hoffman et al. (2013), in that it caused subsequent researchers to attribute deviations from Hoffman et al.’s (2013) published values to other factors only, such as the nature of the corpus (e.g. Hsiao & Nation, 2018).

2 Note that a similar pattern of results was observed using shorter context length (100- instead of 1000-word window; see supplementary Figure 1).
