# Supplementary material for: What is semantic diversity and why does it facilitate visual word recognition?
Source: Behav Res Methods. 2020 Jul 14;53(1):247–63. doi: 10.3758/s13428-020-01440-1 (PMC7880980; doi:10.3758/s13428-020-01440-1)
Supplement: Supplementary file 1 — (DOCX 3581 kb) [file 13428_2020_1440_MOESM1_ESM.docx]

**Supplementary Material**

| **Summary of Results** | | | | | | | | | | | | | | | | | | | | | | | | |
| --- | --- | --- | --- | --- | --- | --- | --- | --- | --- | --- | --- | --- | --- | --- | --- | --- | --- | --- | --- | --- | --- | --- | --- | --- |
|  | **BLP Lexical Decision Reaction Time** | | | | **BLP Lexical Decision Accuracy** | | | | **ELP Lexical Decision Reaction Time** | | | | **ELP Lexical Decision Accuracy** | | | | **ELP Naming**  **Reaction Time** | | | | **ELP Naming**  **Accuracy** | | | |
| *Predictors* | *b* | *SE* | *t* | *p* | *b* | *SE* | *t* | *p* | *b* | *SE* | *t* | *p* | *b* | *SE* | *t* | *p* | *b* | *SE* | *t* | *p* | *b* | *SE* | *t* | *p* |
| SemD | -0.02 | 0.00 | -17.64 | **<0.001** | 1.26 | 0.02 | 11.56 | **<0.001** | -0.02 | 0.00 | -23.78 | **<0.001** | 1.29 | 0.01 | 20.54 | **<0.001** | -0.01 | 0.00 | -15.97 | **<0.001** | 1.13 | 0.01 | 8.30 | **<0.001** |
| CD | -0.08 | 0.00 | -30.30 | **<0.001** | 7.28 | 0.08 | 24.28 | **<0.001** | -0.07 | 0.00 | -28.78 | **<0.001** | 2.82 | 0.05 | 21.44 | **<0.001** | -0.04 | 0.00 | -16.00 | **<0.001** | 1.82 | 0.06 | 10.58 | **<0.001** |
| AoA | 0.05 | 0.00 | 48.76 | **<0.001** | 0.42 | 0.02 | -35.54 | **<0.001** | 0.06 | 0.00 | 64.27 | **<0.001** | 0.40 | 0.02 | -56.93 | **<0.001** | 0.06 | 0.00 | 59.99 | **<0.001** | 0.43 | 0.02 | -45.66 | **<0.001** |
| Length | 0.01 | 0.00 | 11.43 | **<0.001** | 1.51 | 0.02 | 23.54 | **<0.001** | 0.05 | 0.00 | 66.40 | **<0.001** | 1.51 | 0.01 | 33.61 | **<0.001** | 0.05 | 0.00 | 63.54 | **<0.001** | 1.11 | 0.01 | 7.07 | **<0.001** |
| SemD*CD | 0.02 | 0.00 | 15.05 | **<0.001** | 0.62 | 0.04 | -13.19 | **<0.001** | 0.01 | 0.00 | 12.09 | **<0.001** | 0.82 | 0.02 | -8.97 | **<0.001** | 0.01 | 0.00 | 6.42 | **<0.001** | 0.89 | 0.03 | -4.33 | **<0.001** |
| SemD*AoA | 0.01 | 0.00 | 7.72 | **<0.001** | 0.92 | 0.02 | -4.50 | **<0.001** | 0.00 | 0.00 | 4.06 | **<0.001** | 0.97 | 0.01 | -2.15 | **0.031** | 0.00 | 0.00 | 2.85 | **0.004** | 0.99 | 0.02 | -0.74 | 0.459 |
| SemD*Length | -0.00 | 0.00 | -1.72 | 0.086 | 1.05 | 0.02 | 2.84 | **0.004** | 0.00 | 0.00 | 3.60 | **<0.001** | 0.95 | 0.01 | -4.72 | **<0.001** | -0.00 | 0.00 | -2.72 | **0.007** | 0.98 | 0.01 | -1.66 | 0.098 |
| AoA*CD | -0.04 | 0.00 | -23.26 | **<0.001** | 2.71 | 0.05 | 21.55 | **<0.001** | -0.03 | 0.00 | -21.33 | **<0.001** | 1.78 | 0.03 | 20.54 | **<0.001** | -0.02 | 0.00 | -13.84 | **<0.001** | 1.41 | 0.03 | 10.39 | **<0.001** |
| Length*CD | 0.00 | 0.00 | 0.10 | 0.923 | 0.94 | 0.03 | -1.73 | 0.083 | -0.00 | 0.00 | -0.10 | 0.922 | 0.91 | 0.02 | -4.00 | **<0.001** | 0.00 | 0.00 | 2.12 | **0.034** | 0.97 | 0.03 | -0.93 | 0.352 |

**Table 1.** Validation analysis model results where word frequency was replaced with contextual diversity (document count).

| **Simulation Analyses across Corpora and Context Length** | | | | | | | | | | | | | | | |
| --- | --- | --- | --- | --- | --- | --- | --- | --- | --- | --- | --- | --- | --- | --- | --- |
| *Rodd et al. (2002)* | | | | | | | | | | | | | | | |
|  |  | | **100-word window** | | | | | **Wackypedia** | | | | **ukWac** | | | |
| *Predictors* | *df* | | *F* | | *p* | | | *F* | | *p* | | *F* | | *p* | |
| Senses | 121 | | <0.01 | | 0.99 | | | 9.39 | | **<0.01** | | 1.51 | | 0.22 | |
| Meanings | 121 | | 1.07 | | 0.30 | | | 1.33 | | 0.25 | | 0.78 | | 0.38 | |
| *Armstrong & Plaut (2016)* | | | | | | | | | | | | | | | |
|  | **100-word window** | | | | | **Wackypedia** | | | | | **ukWac** | | | | |
| *Predictors* | *b* | *SE* | *t* | *p* | | *b* | *SE* | | *t* | *p* | *b* | *SE* | *t* | | *p* |
| Polysemy | -0.00 | 0.04 | -0.03 | 0.980 | | 0.09 | 0.03 | | 2.78 | **0.006** | 0.04 | 0.03 | 1.29 | | 0.200 |
| Homonymy | -0.02 | 0.02 | -0.99 | 0.321 | | -0.01 | 0.02 | | -0.69 | 0.489 | -0.02 | 0.02 | -1.45 | | 0.150 |

**Table 2.** Results of simulation analyses of Rodd et al. (2002) and Armstrong and Plaut (2016) across corpora and context length.


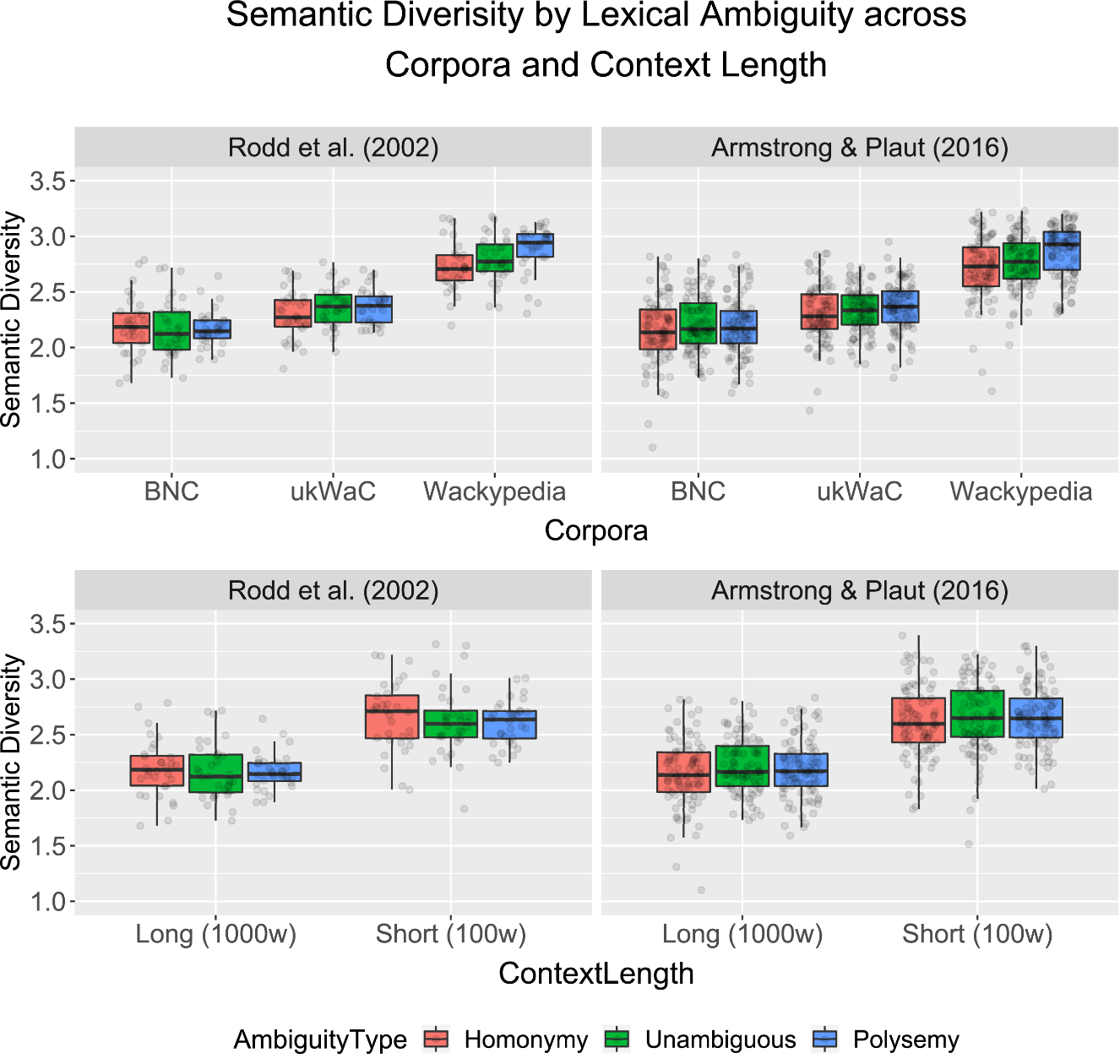


**Figure 1.** Semantic Diversity computed across corpora and context length by lexical ambiguity based on the stimuli from Rodd et al. (2002) and Armstrong and Plaut (2016).


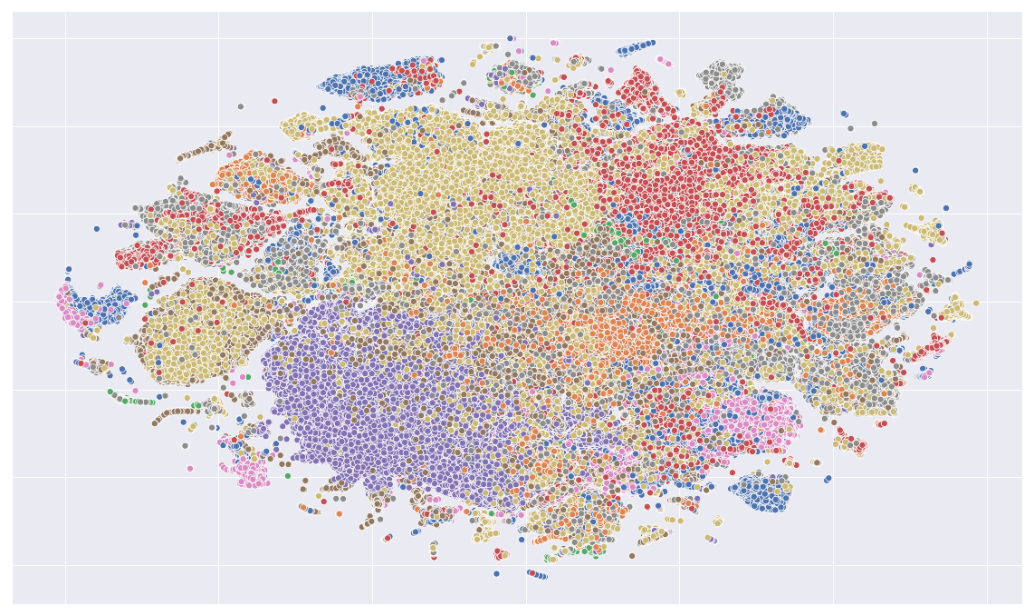

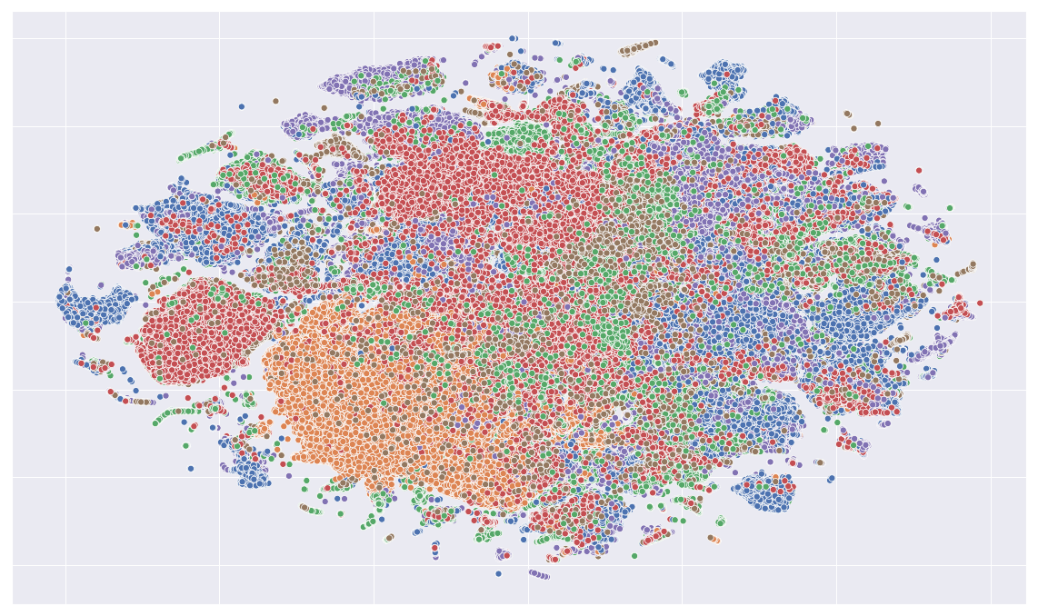


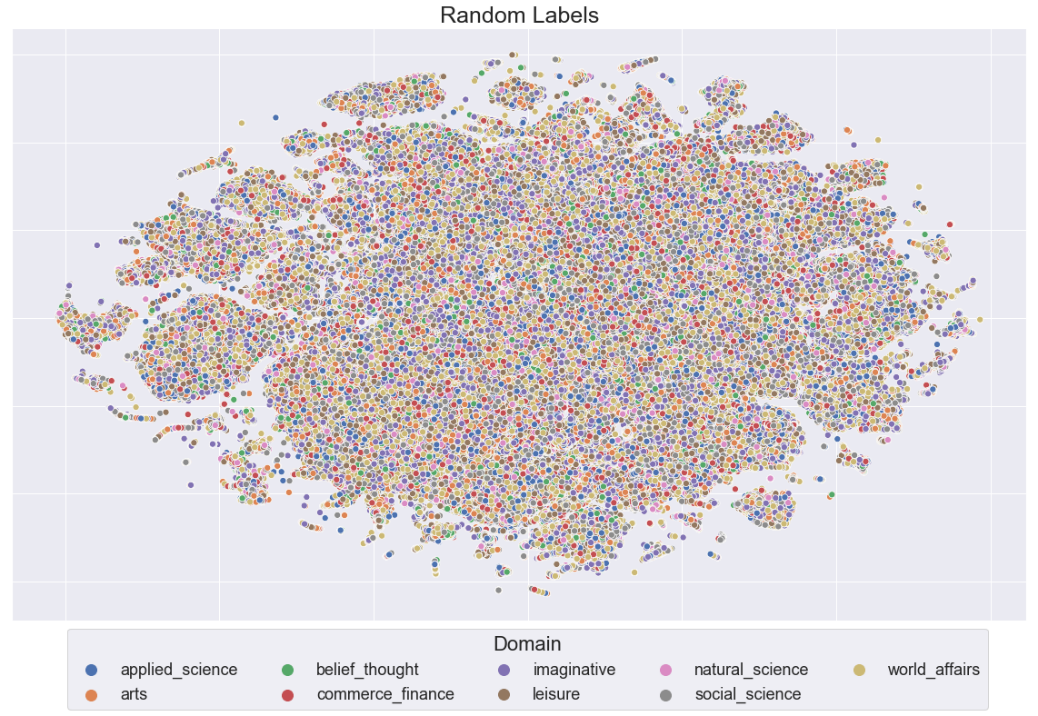

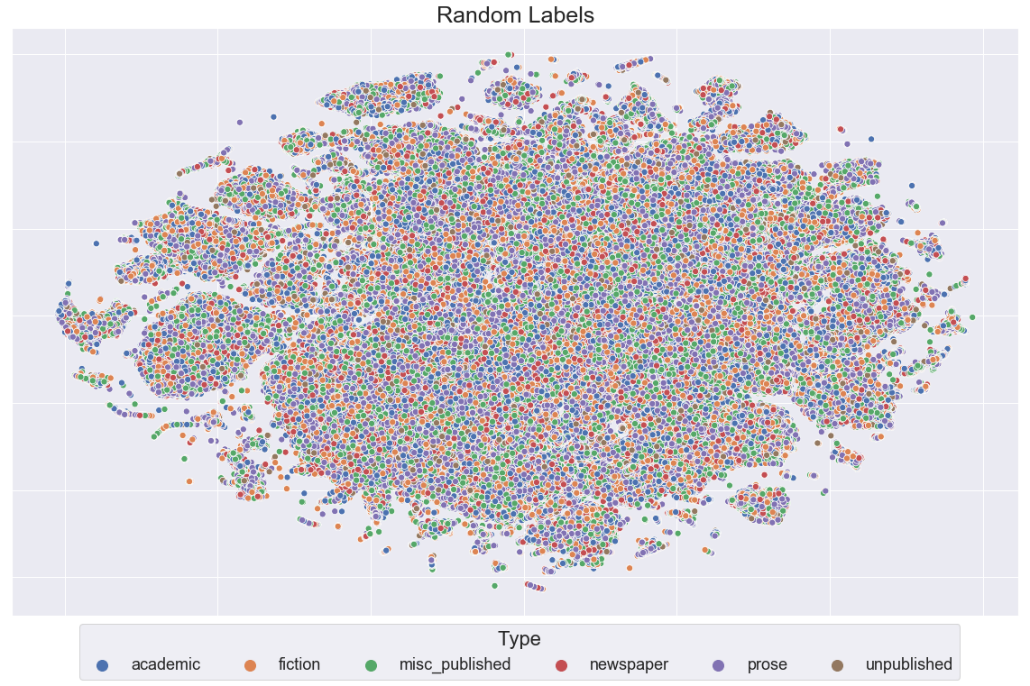


**Figure 2.** t-SNE plots of the whole corpus labelled by domain (left) and type of written text (right) using a 100-word window as context length.
